# A novel model of molnupiravir against SARS-CoV-2 replication: accumulated RNA mutations to induce error catastrophe

**DOI:** 10.1038/s41392-021-00837-4

**Published:** 2021-12-02

**Authors:** Yuxi Zhao, Gu He, Wei Huang

**Affiliations:** 1 West China Hospital of Stomatology, Sichuan University, Chengdu, China; 2 State Key Laboratory of Biotherapy and Cancer Center, West China Hospital, Sichuan University and Collaborative Innovation Center of Biotherapy, Chengdu, China; 3 State Key Laboratory of Southwestern Chinese Medicine Resources, Hospital of Chengdu University of Traditional Chinese Medicine, School of Pharmacy, Chengdu University of Traditional Chinese Medicine, Chengdu, China

**Keywords:** Drug discovery, Chemical biology

**Fig. 1:**
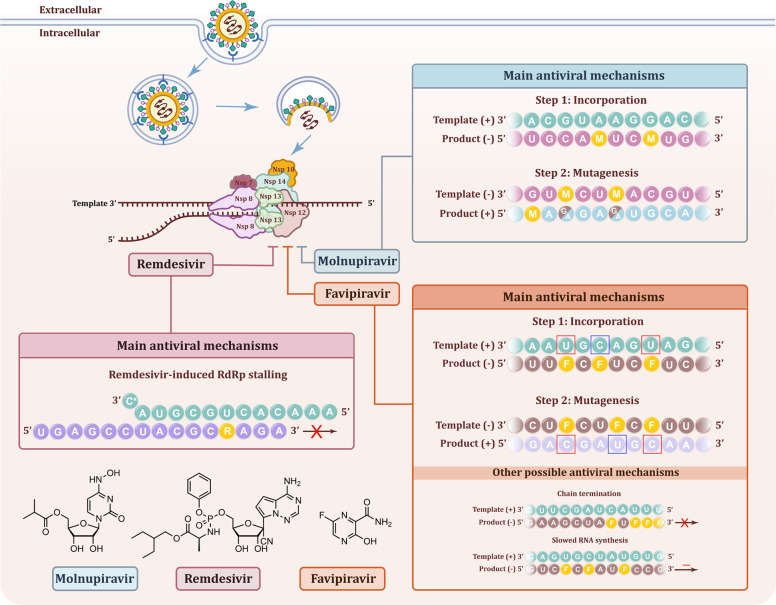
Mechanisms of molnupiravir, remdesivir, and favipiravir in against SARS-CoV-2. The virus binds to the ACE2 receptor of the host cell and then be internalized. The membrane of the vacuole fuses with the virus and release the genome of the virus into the cytoplasm of the host cell. The virus genome translated and produced the RNA replicase-transcriptase complex which contains 16 NSPs. One of the NSPs, non-structural protein 12 (Nsp12) has the function of RdRp and could mediate the replication of viral genome together with other replicases. Molnupiravir, remdesivir, and favipiravir could inhibit the function of RdRp. The mechanisms were as follow: (1) Molnupiravir induced RNA mutagenesis by two steps: M nucleotides can be incorporated by RdRp into the negative-strand genomic (−) during copying of the positive-strand template (+) instead of C or U. The obtained negative-strand RNAs containing M can then be used as a template to produce mutagenized positive-strand. These RNA products are predicted to be mutated and results in nonfunctional viruses formated. RNA of random sequence is shown, with M and mutated residues indicated as yellow and brown letters, respectively. (2) Remdesivir acts as a nucleoside analog and could be incorporated into the growing RNA strand by the RdRp. The replacing of ATP with RTP leads to an elongation barrier after addition of three more nucleotides and induces RdRp stalling. RNA of random sequence is shown, with R indicated as yellow. (3) Favipiravir, a purine nucleic acid analog, could mimics the purines G and A to form non-canonical base pairs and has also been found to act against coronaviruses via other mechanisms, including non-obligate chain termination and slowing of RNA synthesis. RNA of random sequence is shown, with F indicated as yellow. The mutated residues are marked with blue or red boxes

RNA-dependent RNA polymerase (RdRp), also known as nsp12, is a key component in the synthesis of viral ribonucleic acid (RNA) and is considered the primary target of nucleoside analog (NA) inhibitors. A recent publication by Kabinger et al. reported the unique molecular mechanisms of molnupiravir on inducing RNA mutations by RdRp during replication of SARS-CoV-2.^1^ As an oral broad-spectrum NA antiviral agent, molnupiravir has been proven with considerable potential for the prevention and treatment of Corona Virus Disease 2019 (COVID-19) (Fig. 1).^2^

The outbreak of the COVID-19 pandemic has led researchers and clinicians to repurpose existing antiviral NAs to fight SARS-CoV-2 infection. Since RdRp plays a pivotal role in virus replication and transcription, broad-spectrum NA inhibitors have been used to target RdRp, which can recognize and incorporate the active form of NA inhibitors into the growing RNA strand, thereby terminating RNA chain elongation. However, the 3′–5′ exonuclease (ExoN) of SARS-CoV-2 may limit the antiviral effects of NA inhibitors incorporated into nascent RNA.^3^ To date, some NAs which can evade the ExoN proofreading function have shown potent anti-coronavirus activity in preclinical studies and have entered clinical trials for COVID-19 treatment, among which molnupiravir was just approved by Australia on August 9, 2021, for treating COVID-19.

Molnupiravir is an isopropyl ester prodrug of β-d-*N*^4^-hydroxycytidine (NHC), the first mutagenic NA that was demonstrated to circumvent ExoN proofreading and has been identified as a potent inhibitor of murine hepatitis virus (MHV), respiratory syncytial virus (RSV), influenza B virus, influenza A virus and Middle East respiratory syndrome coronavirus (MERS-CoV). Although molnupiravir can inhibit SARS-CoV-2 replication in human lung tissue,^1^ the underlying mechanisms have not yet been clarified. Here we discuss the findings of a recent systematic biochemical analysis that proposed a two-step mechanism for molnupiravir-induced mutagenesis of coronavirus RNA.^2^

RNA elongation assays were first conducted using recombinant SARS-CoV-2 RdRp and four synthetic RNA template–product duplexes. The NHC triphosphate (MTP) incorporation by SARS-CoV-2 RdRp was quantified using a fluorescent label at the 5′ end of the RNA product strand. The results indicated that NHC monophosphate (M) can be readily incorporated into RdRp instead of cytidines cytosine (C) or uracil (U), and can base-pair with purines guanine (G) or adenine (A), which is consistent with the fact observed in MERS-CoV previously. This is similar to how favipiravir induces SARS-CoV-2 RNA mutagenesis.^1^ Nevertheless, the viral RdRp incorporated M into the RNA strand less efficiently than the correct nucleotides C or U. After incorporating NHC, RdRp was able to extend the growing RNA strand by 4 or 11 nucleotides until the end. Thus, molnupiravir shows a completely different mechanism from chain-terminating NAs such as remdesivir.^4^

To clarify the role of M-containing RNA in the next step of RNA synthesis, RNA incorporating the M nucleotide at the templating position +1 was prepared by successive solid-phase synthesis using *N*^4^-hydroxy-*N*^4^-benzoylcytidine 3′-(2-cyanoethyl) diisopropyl phosphoramidite as the substrate. The purity of the resulting RNA oligonucleotide was assessed by denaturing high-performance liquid chromatography and high-resolution electrospray ionization mass spectrometry. The templating properties of NHC were investigated by annealing the M-containing RNA oligo with a fluorescently labeled product RNA. The M residue at position +1 effectively directed the incorporation of G or A into nascent RNA, forming M-GTP or M-ATP base pairs in the RdRp active site. Thermal melting experiments showed that the stability of RNA duplexes containing M–G or M–A base pairs was slightly lower than that of C–G-containing duplexes, which could explain how they can escape the proofreading mechanism. These results suggest that when RdRp uses M-containing RNA as a template, the enzyme can subsequently incorporate a correct or incorrect nucleotide into the growing RNA strand, leading to mutagenesis. This process is previously known as *error catastrophe*.^1^

To elucidate the mechanism of NHC-induced RNA mutagenesis, mutagenic RdRp–RNA complexes were prepared and analyzed by cryo-electron microscopy.^2^ Specifically, M-containing RdRp–RNA complexes with G or A at the 3′ end of the strand were prepared to form RdRp–RNA structures bearing an M–G or M–A base-pair at position −1. The respective structures were solved to overall resolutions of 3.3 or 3.2 Å, while the active site was resolved to a resolution of ~2.9 Å, allowing the characterization of a post-translocation state with a free nucleoside triphosphate (NTP)-binding site at position +1. The conformations of protein and nucleic acid did not differ significantly between the two structures or from the corresponding conformations in the structures of RdRp complexed to wild-type or remdesivir-containing RNA. Modeling of M–A or M–G base pairs into the electron density at the −1 position suggested that the amino tautomer of NHC can mimic C to hydrogen-bond with G, while the imino tautomer can mimic U to hydrogen-bond with A.

Taken together, these experiments suggest a two-step mechanism in which molnupiravir induces SARS-CoV-2 RNA mutagenesis by forming stable M–G and M–A base pairs in the RdRp active site. So far, remdesivir, favipiravir, and molnupiravir are only approved NA drugs for the treatment of COVID-19, and all three can evade the ExoN proofreading function. Specifically, the incorporation of remdesivir allows the addition of three nucleotides and stalls RdRp through competitive inhibition, while favipiravir and molnupiravir participate RNA synthesis, inducing mutagenesis.^4^ Molnupiravir mimics the cytidines C and U, whereas favipiravir mimics the purines G and A to form non-canonical base pairs.^3^ Favipiravir has also been found to act against coronaviruses *via* other mechanisms, including non-obligate chain termination and slowing of RNA synthesis.^3^ By building on the known three-dimensional structure of SARS-CoV-2 RdRp,^5^ mechanistic studies of these drugs should contribute significantly to the discovery of promising drug candidates for SARS-CoV-2.

In summary, mechanism revealed in the currently discussed study by Höbartner and Cramer’s group may help explain and optimize drugs against the current and future coronavirus pandemics. At the same time, drugs that rely on inducing RNA mutations may exert genotoxic effects: molnupiravir-induced mutagenesis has been reported to occur in mammalian cells, indicating the possibility that molnupiravir and its similar drugs may fail due to mutagenic effects.^5^ Therefore, future studies should assess the potential long-term genotoxic side effects of molnupiravir. Future work should also explore the clinical efficacy of molnupiravir against COVID-19, especially in light of new SARS-CoV-2 variants, even though it has been proven effective against several variants in vivo.
